# Medical Society of the County of Albany, Semi-monthly Meeting, Dec. 13th, 1871

**Published:** 1872-01

**Authors:** James S. Bailey


BUFFALO

### Medical and Surgical Journal.

VOL. XI. JANUARY, 1872. No. 6.

### Original Communications.

:o:

ART. I.—*Medical Society of the County of Albany. Semi-Monthly Meeting, Dec. 13th,* 1871. *Reported by* JAMES S. BAILEY, M. D.

Dr. JOSEPH LEWI, President, in the Chair.

Dr. JNO. P. V. QUACKENBUSH, after congratulating the President and making a few complimentary remarks, proceeded to report a case of HYSTEROTOMY.

Mrs. Madison, a deformed dwarf, aged 30, prima-para Nov. 19, supposed she was about to be confined and sent for Dr. Northrop, of this city. The next day being called out of town he notified her it would be impossible for him to attend her, when she sent for a midwife and remained in her charge till the evening of the 22d, when Dr. Quackenbush was called and found the pains frequent and severe. On examination he found the head firmly compressed and trying to enter the superior strait of a deformed pelvis. He at once saw the impossibility of a natural delivery, and assisted by Dr. C. H. Porter he performed craniotomy, but found that the skull, though collapsed, could not be drawn through the contracted strait. Nothing now remained for the woman save the chance which the caesarean operation, performed under unfavorable circumstances, might offer. As the patient was exhausted by the protracted labor and want of rest they thought an opiate would be desirable and gave her 1-2 grain of morphine, which procured her a good night's rest, and she awoke refreshed on the morning of the 23rd. He now proceeded to operate, assisted by Drs. Thomp.

son, Hun, Porter, Boulware, Robertson and Lyon. Dr. Thompson administered the ether, and the patient being duly anesthetized, Dr. Quackenbush made the incision through the abdominal walls from a point one half an inch below the umbilicus to within two inches of the pubis. The wound being laid wide open and the intestines drawn one side, the distended uterus was brought fully in view. An incision four inches in extent was made through the walls through which the foetus was first extracted; the placenta was removed through the same orifice. In making the incision through the uterine walls an artery was severed, and considerable hemorrhage ensued, which conld only be restrained by the introduction of a silver suture which was used with a second one to bring the gaping edges of the wound together, as the uterus remained in a state of inertia. After the cavity had been cleansed the external incision was brought together and kept in position by five deep silver sutures and four hair lip pins twisted in figure of eight style with silk thread. The abdomen was now cleansed with warm water and adhesive straps drawn between each of the sutures and a compress and bandage applied around the body.

The patient now came from under the anæsthetic, appeared well, and only complained that the bandage was rather tight. She remained comfortable during the day. Thursday slept well during the night; remained in the same condition Friday and Friday night. On Saturday morning the case appeared encouraging, but in the afternoon her breathing became laborious and her pulse much accelerated. During the night she became delirious, and died of peritonitis on Sunday morning, having survived the operation seventy-two hours.

The following causes for the unfavorable result are assigned by Dr. Quackenbush:

1st. The long duration of the labor which had continued for ninety-six hours. Prolonged muscular action, not only reduces the strength of the patient, but favors hemorrhage from the uterine incision and the utero-placenta sinuses by the induction of uterine muscular inertia, and consequently the failure of prompt contractions after the removal of the fœtus.

2nd. From the general exhaustion which favors the production of a fatal result from shock, peritonitis and septicaemia.

3d. His waiting to perform craniotomy prior to the caesarean section, which caused the patient additional exhaustion, and,

4th. The necessity of using the sutures to draw together the wound in the uterus, caused by the want of contractability of its tissues.

Dr. Quackenbush remarked, with the light of experience afforded by this case, should he meet with a similar case he would operate promptly and at once, for he thought these cases promised success as much as did those where craniotomy was performed, for in the former we find patients in health, in the latter they undergo exhaustion consequent upon diseased action for one, two, or three days.

Dr. Quackenbush said, having now, Mr. President, given the Society the history of this individual case he would give a brief summary of the case, furnished by Dr. Robert P. Harris, President of the Obstetrical Society, of Philadelphia, his opinion as to the more frequent performance of this operation, &c.

Dr. Quackenbush then read extracts from a letter Dr. Harris had recently sent him. He remarked that Dr. Quackenbush's case was undoubtedly the most recent operation in the United States, and said: "I have found five cases for 1869, but only two for 1870 and one for 1871. No doubt there have been other operations but they have not as yet been reported.

In the next number of the American Journal of Obstetrics will appear an abstract of caesarean cases credited to the United States, many of them almost inaccessible to the profession by reason of the scarcity of the journals in which they have been published. Your case will close this collection. I have found eight English and seven American cases in which uterine sutures were used, twelve of them having occurred during the past seven years, with four recoveries. Your case was, like many other, lost on account of long delay in the hands of a midwife. Had you been called early there would have been no muscular inertia of the uterus, and peritonitis, which is an adynamic inflammation in many cases, and especially favored by exhaustion, might not have recovered.

I am glad to see that operators are becoming less afraid of the employment of uterine sutures, and especially those of silver, cut close and bent down upon the face of the uterus, as these have been found, after death, not to have occasioned any peritonitis. except of a local character sufficient to close them in a deposit of lymph. I believe the longitudinal spiral or interrupted suture of Spencer Wells might be made of considerable value; it certainly did well in his case."

Drs. Craig, Vanderpoel and others participated in an interesting discussion upon the case reported.

Dr. Quackenbush related the history of the case where a negro girl operated upon herself with a case-knife in Nassau village, eighteen miles from this city. She was taken in labor and went behind the barn and performed the caesarean section upon herself, as before mentioned, then returned to the house and sent for a doctor. When he arrived he found her nearly pulseless from the shock and loss of blood. The doctor could get but little from her in reference to her condition, but noticed she held her hand over her abdomen. Upon close scrutiny he observed her clothing bloody, and then immediately discovered the cause. He closed the wound in the usual way and the patient recovered. The attending physician related the case to Dr. Thomas Hun of this city.

Dr. JOSEPH LEWI gave the history of a case of THROMBUS VULVA. On the 7th of October he was called to see a woman that received a fall. Upon arriving at the sick room he learned that she was in her ninth month of pregnancy, and that two hours before she undertook to clean the top of a bureau, to accomplish which she stood with one foot on a wooden chair and the other on the top of a chest. That while dusting she lost her balance, and in her effort to regain herself, the chair turned and she fell astride of it. She was immediately placed in bed her countenance indicated violent pain. At intervals of three or four minutes she appeared as if having labor pains; in the intervals she complained of smarting, cutting pains in the region of the vagina. He beheld a sight after exposing her which he never before observed. In the region of the vagina, between the legs, there was a tumor the size of a new-born child's head, perfectly round in appearance, and of a dark, livid color. The substance was as hard as a foetal head. In trying to ascertain the extent of the injury he found that it was impossible to introduce his finger by the front under the os pubis as well as on the side of the vagina. So he by great exertion reached the os uteri posteriorly, causing much pain to the patient, and was astonished to find the os not dilated in the least. The pulse was 70. Dr. Lewi concluded not to resort to active treatment as recommended by different authorities, and ordered warm water applied to the tumor and prescribed morphine to alleviate the pain and give rest. Dr. James S. Bailey was then sent for in consultation. The expectant course was adopted for the time being. The anodyne had the desired effect and the patient was more comfortable the next morning. The Hæmatocele remained the same in size and consistency, but the color was less dark on the left side and center, but of a very dark color along the edge and extending from two to three inches towards the center. A small quantity of dark fluid oozed from the internal half of the tumor. Pains resembling labor occurred from time to time with more or less violence and had to be controlled by opiates. The urine was passed during the night, and no alarming symptoms being manifest, the same treatment was continued.

On the 3rd day after the accident Dr. Lewi was summoned many times, the woman was supposed to be in labor and near delivery. A careful examination proved that labor had not even begun. The pain was produced by the congestion of the parts. The dark color of the edge of the left labia was reduced to the width of two inches; the consistency of the tumor was just as compact as before, but on the outside a little more elastic. The general symptoms were more favorable, the pulse more regular, the bowels were moved by an injection, and the urine passed voluntarily.

On the morning of the 10th, sixty hours after the accident, the tumor broke on the inside of the vagina, letting out a large quantity of fluid and coagulated blood, diminishing the outside tumor to half its former size, and leaving at the place of rupture a cavity the size of a hen's egg. The patient declared that she was now for the first time, without pain. A very offensive odor filled the room. A mild solution of carbolic acid was used as a wash, continuing the warm applications externally as before, and a nourishing diet was recommended. The hemorrhage continued up to the 11th; the size of the tumor was now one-fourth of its original size. The discoloration was reduced to a narrow strip. The pulse was now almost regular, and the general condition of patient much improved. The functions then were performed naturally and there was a desire for food; anodynes were no longer necessary.

On the 13th premonitory symptoms of labor commenced. The os uteri could be reached without difficulty and without pain to the patient. The dilatation indicated the approach of real labor. On the 17th at noon Dr. Lewi was called, and while preparing for an emergency, he discovered upon examination that the tumor was no impediment to a successful delivery. After two hours she had the pleasure of folding the new born infant in her arms. The application of astringent washes were now discontinued; there was no hemorrhage, no gangrene, and no sloughing followed. The tumor has disappeared and the patient enjoys excellent health.

Dr. Quackenbush then read from the American Journal of Obstetrics a portion of Dr. Barker's lecture upon the subject, and remarked that he had seen two cases. One case occurred in the practice of Dr. Armsby; the tumor was freely incised and the cavity washed with ferri sulphatis. The second case he saw in consultation with Dr. Crounse in the country. This tumor was the size of a foetal head; he applied the forceps and delivered, but the tumor was ruptured and lay in shreds; sloughing took place and the woman died.

Dr. S. O. Vanderpoel then presented a rare anatomical anomaly, a Horseshoe Kidney. The specimen was sent to him by Dr. John P. Gray, of the Utica Asylum. It occurred in a female in a case of paresis. When the autopsy was made, at first the kidney could not be found, but was finally found laying flat upon the spine; instead of the kidneys being united below with a firm band, which is sometimes the case, the union took place above, and the structure was the same throughout, and is undoubtedly congenital. The ureters passed out from the lower third of kidneys on the back side. Paresis is a rare disease in females, some English authorities deny it. Dr. Gray has now two or three cases in the asylum.

The Society then adjourned.

